# Maternal caregivers have confluence of altered cortisol, high reward-driven eating, and worse metabolic health

**DOI:** 10.1371/journal.pone.0216541

**Published:** 2019-05-10

**Authors:** Rachel M. Radin, Ashley E. Mason, Mark L. Laudenslager, Elissa S. Epel

**Affiliations:** 1 Department of Psychiatry, University of California, San Francisco, San Francisco, CA, United States of America; 2 Osher Center for Integrative Medicine, University of California, San Francisco, San Francisco, CA, United States of America; 3 Department of Psychiatry, University of Colorado Anschutz Medical Campus, Aurora, CO, United States of America; Swansea University, UNITED KINGDOM

## Abstract

Animal models have shown that chronic stress increases cortisol, which contributes to overeating of highly palatable food, increased abdominal fat and lower cortisol reactivity. Few studies in humans have simultaneously examined these trajectories. We examined premenopausal women, either mothers of children with a diagnosis of an autism spectrum disorder (*n* = 92) or mothers of neurotypical children (*n* = 91). At baseline and 2-years, we assessed hair cortisol, metabolic health, and reward-based eating. We compared groups cross-sectionally and prospectively, accounting for BMI change. Caregivers, relative to controls, had lower cumulative hair cortisol at each time point, with no decreases over time. Caregivers also had stable levels of poor metabolic functioning and greater reward-based eating across both time points, and evidenced increased abdominal fat prospectively (all *ps* ≤.05), independent of change in BMI. This pattern of findings suggest that individuals under chronic stress, such as caregivers, would benefit from tailored interventions focusing on better regulation of stress and eating in tandem to prevent early onset of metabolic disease, regardless of weight status.

## Introduction

Alterations in hypothalamic-pituitary-adrenal (HPA) axis activity due to chronic stress may play an important role in the development of psychiatric conditions and metabolic dysfunction, with evidence for both hyper- and for hypo-cortisolism, as assessed via serum and salivary cortisol [1]. In addition to altered endocrine activity, chronic stress is associated with greater reward-related eating [2, 3] and greater eating following acutely stressful events [4–7]. Chronic stress may also be associated with worse metabolic health, including greater body mass index (BMI) and waist circumference [2, 7], as well as individual components of the metabolic syndrome [8]. Indeed, animal models have shown that chronic stress prospectively increases cortisol levels, and these increases promote overeating of highly palatable food (“comfort eating”; [9–11]. Over time, such glucocorticoid-stimulated comfort eating contributes to increased abdominal fat and dampened cortisol reactivity to acute stressors [12, 13]. Human models have yet to unpack prospective associations between chronic stress, reward-driven eating, cortisol levels, and changes in abdominal adipose storage and metabolic health. However, such information likely has important clinical implications for the treatment of stress-related eating and subsequent obesity.

In the context of studying chronic stress, the two most commonly measured endocrine measures, serum and salivary cortisol, present several obstacles. For one, these mechanisms are only a snap shot and reflect short-term HPA-axis activity (i.e., minutes, hours, days) [14]. Further, there is a great deal of both intra- and inter-individual variability in salivary cortisol due to circadian rhythm [15], reactivity to transient and chronic stress [16, 17], behavioral confounders (eating, sleep, medication; [18], and measurement error [19]. Thus, there are several potential factors that complicate the interpretation of serum and salivary cortisol.

Assessment of cortisol using hair follicles is a rapidly emerging method that reflects long-term total cortisol exposure (i.e., months to years), and may therefore reflect allostatic load [20, 21]. It is a non-invasive measure, with good intra-individual stability, and reflects free, unbound cortisol [20, 22, 23]. Thus, hair cortisol may be less susceptible to confounds inherent in use of saliva and serum to measure cortisol such as time of sampling as well as participant adherence to collection protocols. Importantly, hair cortisol may be a particularly useful measure when studying conditions involving chronic stress exposure [21, 24, 25].

Preliminary investigations have documented associations between higher hair cortisol and various indices of chronic stress, including chronic pain [26], long-term unemployment [27], self-reported perceived stress in pregnancy [28] and psychological conditions such as post-traumatic stress disorder [29]. Pertinent to the current examination, hair cortisol has been positively associated with BMI [24, 30, 31], waist circumference, waist-to-hip ratio (WHR) [32], metabolic syndrome [32], a history of cardiovascular disease [33] and glycemic control [34]. In fact, hair cortisol may predict acute myocardial infarction in men [35]. Although most existing findings link psychosocial adversity and poor health to higher hair cortisol, some studies report that people with anxiety disorders tend to have lower hair cortisol [36].

Caregiving for a child with an autism spectrum disorder (ASD) is one model of chronic stress warranting of research attention. Compared to parents of neurotypical children, parents of children with ASD report greater levels of subjective distress [37]. Further, parents of children with a serious mental illness have lower daily morning salivary cortisol after stressful days [38]. No studies we are aware of have examined hair cortisol (reflecting accumulation over time), in parents of children with ASD. Given the mixed literature on anxiety, general caregiving stress, and hair cortisol, mothers of a child with ASD may have higher or lower levels of hair cortisol [36, 39]. For example, one study found that high-stress mothers of children with ASD have lower hair cortisol relative to the low-stress mothers of neurotypical children [39].

The primary aims of the current investigation were to understand whether caregivers for a child with ASD, compared to their counterparts caring for a neurotypical child, differed at baseline and in change over two years in aspects of HPA axis function and metabolism function, including: (1) hair cortisol, (2) metabolic health (abdominal fat, insulin sensitivity, and lipid profile), and (3) self-reported drive to overeat (reward-based eating). Further, we assessed whether these cross-sectional and prospective associations were independent of BMI and change in BMI, as caregivers tend to have higher BMI and gain more weight over time compared to controls [40].

## Material and methods

### Participants

Participants were female, premenopausal (age 20 to 50 years), and either (1) mothers of a child with a diagnosis of ASD (*n* = 92; herein referred to as the “caregiver” group), or (2) mothers of neurotypical children (*n* = 91; herein referred to as the “control” group). See Aschbacher et al. (2017) and Prather et al. (2015) for additional participant detail. Participants were matched on age, ethnicity, BMI, and education across groups. All participants had at least one child between the ages of 2 and 16 years, had a body mass index (BMI) <40 kg/m^2^ (thus excluding individuals with extreme obesity), were English-speaking, and did not smoke. Potential participants were excluded if they reported major medical diseases (e.g., history of stroke, heart attack), major psychiatric illnesses (excepting major depressive disorder in caregivers, which is common in caregiving), eating disorders, endocrine disorders (e.g., diabetes, polycystic ovarian syndrome), or treatment that could confound outcome measures (e.g., regular steroid use). Approximately 12% (*n* = 11) of caregivers reported taking antidepressants regularly. Antidepressant usage was unrelated to BMI at any time point (*ps*>.14) and was therefore not included as a covariate in our main analyses. As described elsewhere [41], inclusion criteria for mothers in the caregiver group included caring for a child diagnosed with ASD, and having a minimum Perceived Stress Scale (PSS-10 item) score of 13 upon the initial phone screen, indicating at least a moderate degree of stress. By contrast, women were included as controls if they were caring for a neurotypical child and endorsed a score ≤19 on the PSS. We chose these scores to ensure that the caregiver vs. control groups were different on PSS on average, but to include a wide range of scores that allowed overlap. The mean PSS score for women in nationally representative samples is roughly 16, and an overlap in PSS scores for the two groups was permitted to reduce longitudinal statistical regression to the mean (see (41) for details). We targeted our recruitment of mothers via flyers posted at the UCSF Autism Clinic, and other relevant clinics and community sites, and internet postings, and asked caregiving moms to identify friends who were similar in age to participate.

### Procedures

#### Screening and follow-up visits

Participants attended an outpatient baseline appointment at the UCSF Clinical and Translational Sciences Institute (CTSI) Clinical Research Services. After providing informed consent, participants completed questionnaires assessing demographic and psychosocial factors, assessments of body composition blood pressure, a fasting blood draw, and a hair sampling procedure. We repeated these assessments 2 years later. The UCSF Institutional review board (IRB) approved all aspects of this study. Some participants self-selected to participate in an optional 12-week stress-reduction mindfulness-based intervention (Health and Resiliency Training for Parenting Stress) at 18-months post-baseline. We retained participants who declined participation in the intervention as a control group (over the course of 12 weeks). Therefore, our models that examined measures at the 2 year assessment (*n* = 108) also accounted for intervention status (enrollees, *n* = 48) as a covariate.

### Measures

#### Hair cortisol

Participants submitted a small hair sample (6cm in length), which we analyzed for cortisol levels over the previous 6 months. A trained staff member cut hair at the root from the posterior vertex of the scalp, as close to the scalp as possible, and disguised as best as possible underneath an outer layer of hair. Participants were free to decline this procedure at the visits if they so desired (at the baseline visit, approximately 8% of participants (*n* = 14), and at the 2 year visit, approximately 21% of participants (*n* = 29) either declined or were unable to provide a hair sample of sufficient length or quantity). Hair samples were then stored in aluminum foil as previously described [22]. At each collection time point, we segmented hair samples into a “proximal” 3cm sample, representing the previous 0 to 3 months of cortisol secretion, and a “distal” 3cm sample, representing the previous 4 to 6 months of cortisol secretion. We placed each separate hair sample segment in a pre-weighed 2ml cryovial (Wheaton, Millville, NJ) and washed three times in 100% isopropanol and dried as previously described [22, 42]. Cortisol levels were determined using a commercial high sensitivity EIA kit (Salimetrics LLC, State College, PA) per manufacturer's protocol as described previously [42]. Inter-assay coefficient of variation (CV) for the control hair pool was 9.2% and intra-assay CV was 2.8%. The detection limit was below the limit of sensitivity of the assay if hair weight was ≤5mg [42]. Approximately 4.5% of all collected proximal and distal hair cortisol samples were considered below the detection status due to low mass of hair and were therefore excluded from all analyses. Thus, at baseline, a total of 166 participants provided hair cortisol samples above the detection limit, and at the 2-year assessment, 108 participants provided hair cortisol above the detection limit. With regard to attrition of the 2 years, of the initial 166 participant samples provided, 65% were also provided at the 2-year assessment. This decrease was partially due to natural attrition throughout the course of the study, in addition to insufficient quality of sample provided, as well as changes in sampling procedures at the 2 year time point.

#### Hair hygiene questionnaire

We asked participants to complete a brief questionnaire at the 2-year time point regarding variables with potential influence on hair cortisol concentrations (e.g., presence and frequency of chemical straightening, bleaching, coloring, and highlighting of the hair, along with the use of heat treatments and scalp medication).

#### Physiological measures and BMI

We measured resting blood pressure (systolic (SBP) and diastolic (DBP) blood pressure, mm Hg), having participants sit and rest for 5 minutes before and between measures, height by stadiometer (cm) against the wall, and weight by calibrated digital scale (kg). We calculated body mass index (BMI) as weight in kilograms divided by the square of height in meters (kg/m^2^), and percent body fat and lean mass were estimated by the digital scale.

#### Body fat distribution

We measured body fat distribution, including waist circumference (cm) and waist-to-hip ratio (WHR), using a retractable tape measure. Measures were taken twice and averaged together. We measured visceral and trunk fat (“fat mass”) via Duel Frequency Bioelectrical Impendence Analysis (BIA) technology using a Tanita Professional ViScan (AB-140). Visceral and trunk fat were statistically estimated using validated formulas, where the Tanita values were highly correlated with assessments from CT scans and DEXA scans.

#### Phlebotomy

A registered nurse collected up to 200 mls of blood from participants using a butterfly needle or IV catheter to assess for a variety of metabolic indicators, including cholesterol (total, LDL, and HDL), glucose, insulin, triglycerides, and appetite-regulating hormones, including leptin and ghrelin.

#### Metabolic Syndrome

We defined metabolic syndrome, and its individual components, using sex- and ethnicity- specific cut-off definitions by the International Diabetes Federation (IDF) worldwide consensus guidelines [43], using the above measures (blood pressure, body fat distribution, blood values of lipids and insulin sensitivity; See S1 Table).

#### Eating behavior

The Reward-based Eating Drive (RED-9) scale is a 9-item self-report measure of reward-driven eating and captures a lack of satiety, preoccupation with eating, and loss of control over eating [3]. Items are answered on a Likert scale from 0 (not at all like me) to 4 (very much like me). We computed scores as the mean of all items [44]. Scale reliability was high (α = 0.94).

#### Psychological functioning

The following psychological variables were used in order to evaluate their association with caregiver status, as well as their potential moderating role in the association between caregiver status and hair cortisol: (1) The Perceived Stress Scale (PSS; Cohen et al., 1983) is a 10-item self-report questionnaire that measures a persons’ evaluation of the life stress they have experienced over the previous month. Scale reliability was high (α = .87). (2) The Parental Stress Scale [45] contains 18 items representing positive themes of parenthood (emotional benefits, self-enrichment, personal development) and negative components (demands on resources, opportunity costs and restrictions). Each item is rated on a five-point scale: strongly disagree (1), disagree (2), undecided (3), agree (4), and strongly agree (5), and the positive items are reverse coded so that total or average reflects extent of parenting stress. Analyses suggest the Parental Stress Scale has high internal reliability, and predictive and convergent validity [45]. Scale reliability was high (α = .87). (3) The Inventory of Depressive Symptoms (IDS; [46] is a 30-item questionnaire that screens for depressive symptoms and severity. Scale reliability was high (α = .82). (4) Additionally, a diagnosis current or past major depressive disorder (MDD) was assessed with the Structured Clinical Interview for DSM-IV-TR (SCID) [47]. (5) Finally, the Current Stressors Checklist, a measure created specifically for the current study, is an inventory of life stressors in the past year across 6 domains of life. Responses within each domain are recorded into 3 component items. The first component contains the presence/absence of the stressor. If selected/checked, the second component contains a text description and the third component contains a severity a rating of perceived stress. Furthermore, we collected information regarding number of children in the household with and without ASD.

### Statistical analysis

#### Data preparation

Due to significant skew (proximal: 7.61, distal: 5.86), we log-transformed all hair cortisol values to approximate a normal distribution (proximal: 1.12, distal: 0.59).

We compared caregivers to controls on all demographic and psychological variables at baseline (e.g., age, race, ethnicity, income, education, weight status, number of children, perceived stress, parental stress, IDS) and on all hair hygiene variables (e.g., hair coloring) collected at 24 months, using independent samples t-tests or chi-squared analyses. We coded controls and caregivers as (0,1), respectively, for all analyses.

**Testing group differences in cumulative (hair) cortisol levels at baseline, 2 years later, and in change over 2 years.** In cross-sectional analyses, we compared caregivers and controls (IV: group) on hair cortisol (DVs: proximal and distal 3 cm segments) at baseline and 2 years (four total segments/participant), before and after adjusting for BMI at baseline and 2 years (covariate), respectively, using one way analysis of covariance (ANCOVA). In prospective analyses using separate ANCOVA models, we compared groups (IV) on change in hair cortisol (DV: 2 year minus baseline hair cortisol), adjusting for change in BMI (covariate: 2 year-baseline BMI). This adjustment for change in BMI allowed us to answer the question of whether caregivers have greater changes in hair cortisol (increase, or decrease), independent of BMI change. We ran an identical model at 2 years adjusting for intervention status (intervention vs. control).

**Testing group differences in metabolic health at baseline, 2 years, and in change over 2 years.** In cross-sectional analyses, we compared caregivers and controls (IV: Group) along all continuous metabolic factors (DVs: waist circumference, WHR, visceral fat, fat mass, cholesterol, triglycerides, blood pressure, leptin, ghrelin), at baseline and 2 years, before and after adjusting for BMI and/or fat mass (covariate; at baseline or 2 years, respectively), using one-way analysis of covariance (ANCOVA). We compared groups using the categorical variables based on metabolic syndrome cut-off criteria using chi-squared analyses. In prospective analyses using separate ANCOVA models, we compared groups (IV) on change in these same continuous metabolic outcome variables (DV: 2 year minus baseline metabolic variables), adjusting for change in BMI (covariate: 2 year-baseline BMI) or change in fat mass (covariate: 2 year-baseline fat mass). This adjustment for change in BMI and fat mass allowed us to answer the question of whether caregivers have greater changes in metabolic health (increases, or decreases), independent of BMI or fat mass change. We ran an identical model at 2 years adjusting for intervention status (intervention vs. control).

**Testing group differences in eating behaviors at baseline, 2 years, and in change over 2 years.** In cross-sectional analyses, we compared groups on eating-related factors measured at baseline and 2 years (RED-9), before and after adjusting for BMI at baseline and 2 years, respectively, using one-way analysis of covariance (ANCOVA). In prospective analyses using a separate ANCOVA model, we compared caregivers and controls (IV: group) on change in RED-9 (DV1: 2 year minus baseline RED-9), adjusting for change in BMI (covariate: 2 year-baseline BMI). This adjustment for change in BMI allowed us to answer the question of whether caregivers have greater changes in eating behavior (increases, or decreases), independent of BMI change. We ran an identical model at 2 years adjusting for intervention status (intervention vs. control).

In all analyses, we considered differences between groups significant when *p* values were ≤.05. All tests were two-tailed.

## Results

### Participant characteristics

Most participants (age 42.4 ± 5.1 y) identified as non-Hispanic White (76%) and were not overweight (56% below a BMI of 25 kg/m^2^). By study design, caregivers and controls did not differ in BMI, weight status, age, race, ethnicity, or years of education. Additionally, by study design, caregivers reported significantly higher scores on the PSS and were significantly more likely to have a current or past diagnosis of major depressive disorder (MDD). The majority (~80%) of participants had 1 to 2 children, and caregivers and controls did not differ with regard to total number of children. Among caregivers, the majority (91%) had one child with a current diagnosis of ASD, with 8% of caregivers reporting having 2 or more children with a current diagnosis. The average duration of caregiving (from initial diagnosis of a child with ASD) was approximately 5 years (SD = 2.91 years). With regard to annual household income, caregivers generally reported lower income although the sample was in general highly educated and middle class (*p* = .05; Table 1).

**Table 1:** Demographic and anthropometric characteristics of study participants at baseline.

Variable	Total Sample	Caregivers^b^	Controls^c^	*T* or *χ*^*2*^
*n*	183	92	91	
Age (y)^a^	42.4 ± 5.1 (24–50)	42.8 ± 5.6	42.1 ± 4.5	0.93
Race/Ethnicity:				
% Non-Hispanic White	76	76	76	2.09
Annual Household Income, % (*n*):				7.82*
< $100,000	23.6% (43)	31.9% (29)	15.4% (14)	
$100,000-$149,000	27.5% (50)	25.3% (23)	29.7% (27)	
$150,000-$199,000	20.3% (37)	15.4% (14)	25.3% (23)	
≥$200,000	28.6% (52)	27.5% (25)	29.7% (27)	
Education (%):				8.32
< College degree	13.40%	17.80%	9%	
Bachelor’s degree	37.40%	40%	34.80%	
Graduate Degree	49.10%	42.20%	56.20%	
BMI (kg/m2)^a^	25.5 ± 5.2 (17–45)	25.9 ± 5.8	25.1 ± 4.7	1.02
Weight Status (%):				4.99
Non-Overweight	56.40%	52.20%	60.40%	
Overweight	26.50%	30%	23.10%	
Obese	17.10%	17.80%	16.50%	
Perceived Stress Scale (PSS)^a^	18.8 ± 5.5 (7–33)	21.9 ± 4.7	15.7 ± 4.4	9.20**
Parental Stress Scale (mean)^a^	2.34 ± 0.54 (1.17–3.83)	2.64 ± 0.49	2.07 ± 0.42	8.35**
Current Stressors (event count)^a^	1.27 ± 1.16 (0–5)	1.50 ± 1.12	1.03 ± 1.15	2.76**
Inventory of Depressive Symptoms ^a^	15.61 ± 8.07 (3–44)	19.30 ± 8.34	11.96 ± 5.88	6.84**
Current Diagnosis of MDD (%)^d^:				11.60**
Full MDD	4.40%	9.10%	0%	
Subthreshold MDD	1.60%	3.40%	0%	
Previous Diagnosis of MDD (%)^d^:				6.40*
Full MDD	35.00%	27.60%	46%	
Subthreshold MDD	7.10%	8.00%	7%	
Number of children^a^:	1.91 ± 0.83 (1–7)	2.02 ± 0.91	1.80 ± 0.73	1.79^†^
1 child	31.10%	26.10%	36.30%	3.4
2 children	51.40%	53.30%	49.50%	
3 children	14.20%	16.30%	12.10%	
4+ children	3.22%	4.30%	2.20%	
Number of children with ASD^a^:	0.55 ± 0.58 (0–2)	1.09 ± 0.28 (1–2)	0	36.80**
0 children	49.70%	0%	100%	183.00**
1 child	45.90%	91.30%	0%	
2 or more	4.40%	8.70%	0%	
**Hair hygiene questions (n = 114)**				
Chemical Hair Straightening (%)	1.8	1.8	1.7	0.001
Hair Coloring (%)	41.1	50	32.8	3.44^†^
Hair Bleaching (%)	2.7	3.6	1.8	0.38
Hair Highlighting (%)	23	27	19	1.1
Heat Treatment (%)	54.9	53.6	56.1	0.08
Presence of any hair treatment (coloring, heat)	25.7	30.9	20.7	1.55

Note.

*p ≤ .05

**p ≤ .01

^†^trend-level significance.

^a^*M ± SD* (Range)

^b^Caregivers, defined as total PSS score ≥ 13, and caregiver of a child (age 2–16) with a pervasive developmental disorder (e.g., ASD)

^c^Controls, defined as total PSS score ≤ 19, and caregiver of a neurotypical child (age 2–16)

^d^MDD = major depressive disorder.

Caregivers were more generally “stressed” on trait- and state-level self-report measures in comparison to controls. Specifically, caregivers, reported higher scores on the Parental Stress Scale and the Inventory of Depressive Symptoms (IDS), and a greater number of current stressful events (*ps*≤ .01; Table 1). Thus, our caregiver grouping captured the expected higher levels of parental distress, as well as general distress and depressive symptoms.

Caregivers and controls did not differ in terms of hair hygiene (e.g., presence and frequency of chemical straightening, bleaching, coloring, and highlighting of the hair), thus, this we did not include this variable as a covariate in subsequent analyses (Table 1). Further, caregivers and controls did not differ in BMI at the 2 year assessment (caregivers: 25.91 vs. controls: 25.11 kg/m^2^, *p* = .37) nor did they show differential increases in BMI from baseline to 2 years (*F*(1,131) = .009, *p* = .93). Caregivers showed an average increase in BMI of 0.28 kg/m^2^ and controls showed an average increase of 0.36 kg/m^2^.

### Caregiver vs. control differences in hair cortisol

**Fig 1:**
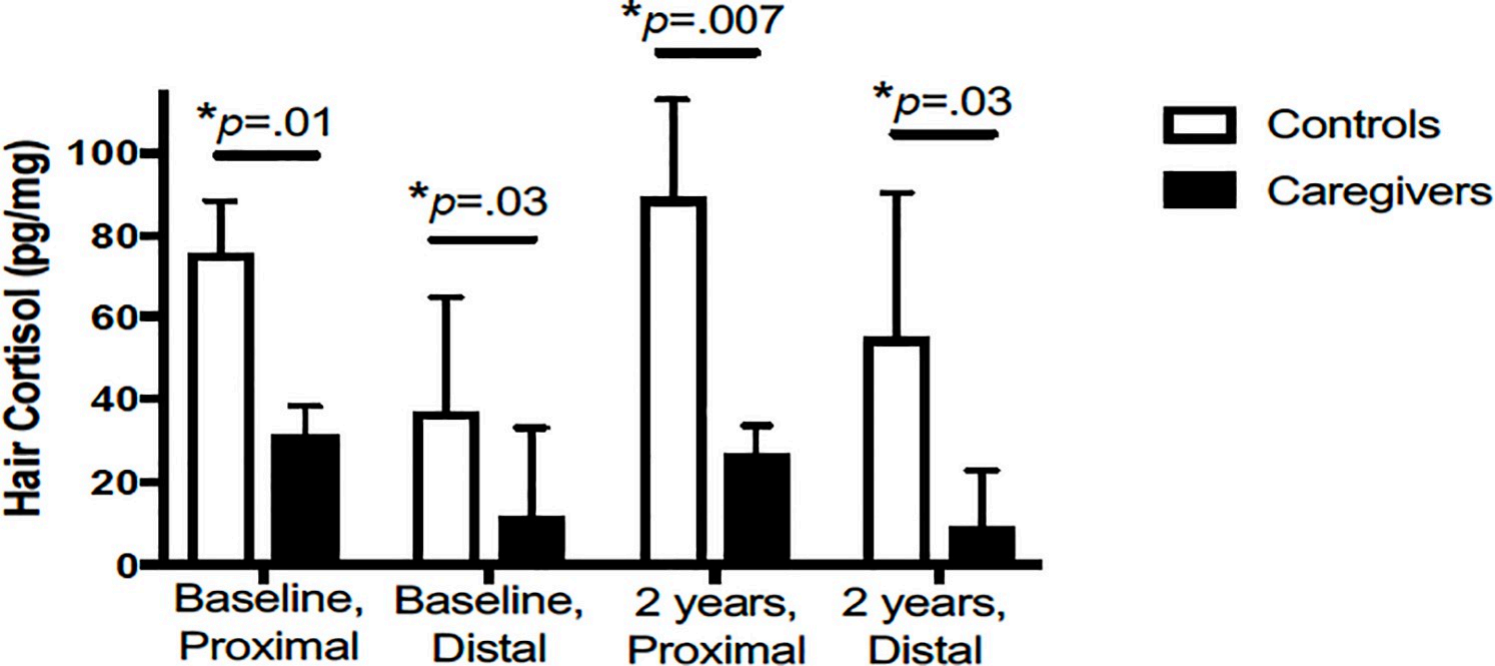
Group differences in hair cortisol at baseline and 2 years. Baseline data are presented from analysis of analysis of covariance (ANCOVA) adjusted for baseline BMI (proximal segment: *F*(1,163) = 6.81, *p* = .01; distal segment: F(1,154) = 4.65, *p* = .03). Two year data are presented from ANCOVA adjusted for 2 year BMI (proximal segment: *F*(1,105) = 7.58, *p* = .007; distal segment: *F*(1,102) = 9.33, *p* = .03). The raw (non log-transformed) adjusted *mean+SE* for hair cortisol is shown for caregiver (filled bar) and controls (open bar) at each time assessment.

Caregivers (*n* = 86) had significantly lower levels of hair cortisol (proximal M±SD: 31.62 ±6.76 pg/mg; distal: 11.75±21.38 pg/mg) compared to controls (*n* = 80; proximal: 75.86±12.59 pg/mg; distal: 37.15±27.54 pg/mg). Indeed, there was a main effect of group for both baseline proximal segment (*F*(1,163) = 6.81, *p* = .01) and distal segment of hair cortisol level (*F*(1,154) = 4.65, *p* = .03). We observed an identical pattern of findings when examining hair cortisol at the 2 year assessment, such that caregivers (*n* = 51) had significantly lower levels of hair cortisol (proximal: 26.91±6.76 pg/mg; distal: 9.33±13.49 pg/mg) compared to controls (*n* = 57; proximal: 89.13±13.80 pg/mg; distal: 54.95±35.48 pg/mg). Indeed, there was a main effect of group for both 2 year proximal segment (*F*(1,105) = 7.58, *p* = .007) and distal segment of hair cortisol level (*F*(1,102) = 9.33, *p* = .03; Fig 1).

In prospective analyses, caregivers did not differ from controls with regard to change in proximal hair cortisol (*p* = .76; Table 2, top panel). On average, caregivers decreased in proximal hair cortisol from baseline to 2 years by 0.95 pg/mg, whereas controls increased in proximal hair cortisol by 1.12 pg/mg, however, these group differences were not statistically significant. Further, when including intervention status in the model, we found no main effect of group, intervention status, or group X intervention status interaction (*ps*>.48). Furthermore, in prospective analyses, caregivers did not differ from controls with regard to change in distal hair cortisol (*p* = .50; Table 2, bottom panel), although there was an average decrease observed among caregivers of 0.90 pg/mg whereas controls evidenced an average increase of 1.56 pg/mg over the 2 years. When including intervention status in the model, we found no main effect of group, intervention status, or group X intervention status interaction (*ps*>.60).

**Table 2:** One-way ANCOVA for group differences in hair cortisol change from baseline to 2 years, adjusting for change in BMI from baseline to 2 years.

	*F*	*p*	*M±SE*_*difference*_	*M±SE*_*difference*_
			Caregivers (*n* = 50)	Controls (*n* = 54)
Main effects:				
Δ Hair Cortisol, proximal (pg/mg)	0.10	.76	-0.95 ± 1.46	+1.12 ± 1.41
Covariates:				
Δ BMI	0.56	.46		
Main effects:				
ΔHair Cortisol, distal (pg/mg)	0.47	.50	-0.90 ± 1.79	+1.56 ± 1.73
Covariates:				
Δ BMI	0.27	.61		

*Note*. Unadjusted analyses not presented, as direction and level of significance remained the same in analyses unadjusted for BMI.

#### Exploratory analyses

In an effort to understand the observed cross-sectional group differences in hair cortisol at each time point, we ran a series of exploratory analyses, first examining correlations between hair cortisol and all study variables of interest at baseline, and second, examining moderating role of psychological factors in the association between group and hair cortisol.

Hair cortisol (both proximal and distal) at baseline was unrelated to any measure of self-reported stress at baseline: PSS, parental stress scale, current stressors, IDS (all *ps*>.09). Furthermore, hair cortisol at baseline was unrelated to any metabolic measure at baseline: insulin, glucose, cholesterol (total, HDL, LDL), trigylcerides, SBP/DBP, waist circumference, BMI (all *ps*>.10; Table 3). Furthermore, a current or past diagnosis of MDD (*n* = 8) was unrelated to hair cortisol at baseline (*ps>*.30) or at 2 years (*ps>*.12), or changes in hair cortisol at 2 years (*ps*>.16) , which contrasts with prior data [20]. However, consistent with prior data [48], a diagnosis of MDD (*n* = 8) was associated with a greater likelihood of obesity status (*p* = .02), elevated systolic and diastolic blood pressure (*ps* = .01), and full metabolic syndrome (*p* = .04) at baseline. However, we did not find prospective support for MDD status predicting worsening of metabolic characteristics at 2 years (e.g., changes in weight, lipids, blood pressure, waist circumference) among our study sample (*ps*>.09).

**Table 3:** Correlations among study variables at baseline.

	1	2	3	4	5	6	7	8	9	10	11	12	13	14	15	16	17	18	19	20
1. Group	—																			
2. PSS	0.57^**^	—																		
3. PaSS	0.53^**^	0.58^**^	—																	
4. CSC	0.20^**^	0.40^**^	0.25^*^	—																
5. IDS	0.46^**^	0.72^**^	0.49^*^	0.41^**^	—															
6. HCCp	-.20^**^	-.12	-.13	-.08	-.09	—														
7. HCCd	-0.18^*^	-.09	-.09	-.02	0.01	0.88^*^	—													
8. Insulin	0.33^**^	0.27^*^	0.21^*^	0.17^*^	0.29^*^	-.05	-.07	—												
9. Gluc	0.06	0.19^*^	0.08	0.12	0.18^*^	-.09	-.08	0.21^*^	—											
10. Chol	-0.01	-.05	-.11	-.04	0.06	0.05	0.06	-.05	-.03	—										
11. HDL	-.24^**^	-.15^*^	-.15^*^	-.16^*^	.23^**^	0.04	0.05	-.47^*^	-.14^†^	0.38^**^	—									
12. LDL	0.08	-.02	-.06	0.03	0.07	0.92	0.01	0.07	0.08	0.87^**^	-0.04	—								
13. TGL	0.15^*^	0.17^*^	0.07	0.06	0.27^*^	0.08	0.06	0.49^*^	-.06	0.26^**^	-.39^*^	0.19^*^	—							
14. Leptin	0.15^*^	0.15^*^	0.03	0.19^*^	0.14^†^	-.06	-.12	0.58^*^	0.19^*^	0.19^*^	.25^**^	0.27^*^	0.31^*^	—						
15. Ghrelin	-0.19^*^	-.06	-0.08	-.01	-.08	0.22	0.10	.41^**^	-.09	0.08	0.28^*^	-.01	-.20^*^	-.22^*^	—					
16. Fat Mass	0.10	0.08	-.02	0.17^*^	0.12	-.04	-.14	0.54^*^	0.16^*^	0.14^†^	-.28^*^	0.24^*^	0.30^*^	0.77^*^	-.23^*^	—				
17. SBP	0.09	0.09	-.02	0.08	0.15^*^	0.29	**-.17**	0.25^*^	0.27^*^	0.12	-.12^†^	0.16^*^	0.15^*^	0.30^*^	-.09	0.37^*^	—			
18. DBP	0.07	0.01	-.05	0.03	0.16^*^	-.01	-.02	0.22^*^	0.19^*^	0.04	-.15^*^	0.10	0.12	0.21^*^	-.08	0.27^*^	0.75^*^	—		
19. WC	0.06	0.10	-.01	0.21^*^	0.14^†^	0.01	-.07	0.57^*^	0.24^*^	0.12	-.38^*^	0.26^*^	0.36^*^	0.78^*^	-.21^*^	0.90^*^	0.40^*^	0.28^*^	—	
20. WHR	0.10	0.09	0.07	0.16^*^	0.13^†^	0.08	0.02	0.51^*^	0.14^†^	0.11	-.38^*^	0.24^*^	0.39^*^	0.51^*^	-.14^†^	0.59^*^	0.30^*^	0.23^*^	0.79^*^	—
21. BMI	0.04	0.10	-.03	0.19^*^	0.13^†^	0.03	-.07	0.59^*^	0.27^*^	0.14^†^	-.33^*^	0.25^*^	0.34^*^	0.80^*^	-.26^*^	0.91^*^	0.44^*^	0.33^*^	0.91^*^	0.58^*^

Note.

**p ≤.01

*p ≤.05

^†^*p* ≤.10. Abbreviations: PSS = Perceived Stress Scale, PaSS = Parental Stress Scale, CSC = Current Stressors Checklist, IDS = Inventory of Depressive Symptoms, HCCp = Proximal hair cortisol, HCCd = Distal hair cortisol, Gluc = Fasting glucose, Chol = Total cholesterol, HDL = High density lipoprotetin cholesterol, LDL = Low density lipoprotein cholesterol, TGL = triglycerides, SBP = systolic blood pressure, DBP = diastolic blood pressure, WC = waist circumference, WHR = waist to hip ratio, BMI = Body mass index

In a series of moderation analyses at baseline (where each psychological/stress-related variable was interacted with group on the association with baseline hair cortisol), we found no main or interaction effects (group X variable of interest) on baseline hair cortisol for the following variables: depressive symptoms (IDS, *ps>*.10), current stressors (*ps>*.16), presence of MDD (*ps>*.15), or number of children in the household (*ps>*.25). However, we did observe a significant interaction effect for the parental stress scale (interaction term p-value = .02 for proximal hair cortisol at baseline) and for SES (interaction term *p*-value = .03 for proximal hair cortisol at baseline; no main effect of SES observed). We observed a trend-level (not statistically significant) pattern among caregivers of higher levels of parenting stress being associated with higher proximal hair cortisol values (*p* = .07), whereas among controls, there was no association between parenting stress and hair cortisol. Among controls, higher levels of SES were associated with higher proximal hair cortisol values. In contrast, among caregivers, there was no association between SES and hair cortisol.

### Caregiver vs. control differences in metabolic health

When comparing caregiver and control participants on continuous measures of metabolic health at baseline, caregivers had greater levels of insulin (*p* < .001) and leptin (*p* = .03), lower levels of HDL cholesterol (*p* = .003) and ghrelin (*p* = .02), and tended to have higher triglycerides (*p* = .08; Table 4, top panel). The direction and significance of these findings was identical when adjusting for baseline fat mass rather than BMI. Further, a greater proportion of caregivers, compared controls, met clinical cut-off criteria for low HDL cholesterol (*p* = .007) and significant insulin resistance (*p* = .006; Table 5, top panel). Caregivers and controls, however, were indistinguishable with regard to all other continuous and clinical cut-off metabolic variables (all *ps*>.21).

**Table 4:** Group differences in metabolic health (continuous variables) at baseline, adjusting for baseline BMI (top panel); and 2 years, adjusting for 2 year BMI (bottom panel).

Variable	Caregivers	95% CI	Controls	95% CI	*df*, *error*	F	p
	(Adjusted M±SE)		(Adjusted M±SE)				
**Baseline**							
Insulin (μU/ml)	15.05 ± 0.53	(14.0, 16.1)	11.26 ± 0.53	(10.22, 12.31)	1, 174	25.82	< .001**
Leptin (ng/ml)	21.21 ± 0.91	(19.4, 23.0)	18.29 ± 0.91	(16.49, 20.09)	1, 174	5.12	.03*
HDL Cholesterol (ng/ml)	56.58 ± 1.5	(53.6, 59.5)	62.89 ± 1.49	(59.95, 65.84)	1, 174	8.89	.003**
Ghrelin (pg/ml)	279.98 ± 14.7	(251, 309)	330.02 ± 14.8	(300.9, 359.2)	1, 174	5.76	.02*
Triglycerides (ng/ml)	84.03 ± 4.32	(75.5, 92.6)	73.24 ± 4.30	(64.76, 81.73)	1, 174	3.12	.08^†^
Fat Mass (%)	24.22 ± 0.50	(22.2, 25.2)	23.57 ± .50	(22.6, 24.6)	1, 178	0.85	.36
SBP (mmHg)	113.43 ± 1.30	(111, 116)	111.83 ± 1.29	(109.3, 114.4)	1, 178	0.77	.38
DBP (mmHg)	68.79 ± 1.08	(66.7, 70.9)	67.83 ± 1.07	(65.7, 70.0)	1, 178	0.4	.53
Waist Circumference (cm)	90.91 ± 0.61	(89.7, 92.1)	91.15 ± .61	(89.95, 92.34)	1, 178	0.08	.78
WHR	0.89 ± 0.008	(0.88, 0.91)	.89 ± .008	(.87, .90)	1, 178	0.67	.42
Total Cholesterol (ng/ml)	173.60 ± 3.04	(167.6, 180)	174.90 ± 3.02	(168.9, 180.9)	1, 174	0.09	.76
LDL Cholesterol (ng/ml)	100.23 ± 2.46	(95.4, 105.1)	97.38 ± 2.44	(92.6, 102.2)	1, 174	0.68	.41
**2 years**							
Insulin (μU/ml)	16.12 ± 0.69	(14.8, 17.5)	12.43 ± 0.65	(11.1, 13.7)	1, 126	15.06	< .001**
Leptin (ng/ml)	23.09 ± 1.05	(21.0, 25.2)	21.06 ± 0.99	(19.1, 23.0)	1, 126	1.97	.16
HDL Cholesterol (ng/ml)	60.29 ± 1.78	(56.8, 63.8)	65.96 ± 1.69	(62.62, 69.30)	1, 126	5.32	.02*
Ghrelin (pg/ml)	298.43 ± 16.2	(267, 330.4)	330.1 ± 15.3	(299.8, 360.4)	1, 126	2.02	.16
Triglycerides (ng/ml)	87.68 ± 6.2	(75.5, 99.9)	75.4 ± 5.9	(63.8, 87.0)	1, 126	2.08	.15
Fat Mass (%)	24.43 ± 0.47	(23.5, 25.4)	23.8 ± 0.45	(22.9, 24.7)	1, 132	1.03	.31
SBP (mmHg)	110.8 ± 3.4	(104.1, 118)	110.9 ± 3.2	(104.6, 117.2)	1, 132	0	.99
DBP (mmHg)	64.42 ± 2.65	(59.2, 69.7)	65.09 ± 2.48	(60.2, 70.0)	1, 132	0.04	.85
Waist Circumference (cm)	93.95 ± 0.69	(92.6, 95.3)	90.94 ± 0.65	(89.7. 92.2)	1, 133	10.11	.002**
WHR	0.90 ± 0.01	(0.88, 0.91)	0.88 ± 0.01	(0.87, 0.89)	1, 133	3.2	.08^†^
Total Cholesterol (ng/ml)	183.16 ± 4.03	(175.2, 191)	177.7 ± 3.7	(170.4, 185.1)	1, 133	0.98	.32
LDL Cholesterol (ng/ml)	113.76 ± 3.52	(106.8, 121)	101.0 ± 3.31	(94.5, 107.6)	1, 125	6.97	.009**

*Note*. Unadjusted analyses not presented, as direction and level of significance remained the same in analyses unadjusted for BMI.

*p ≤ .05

**p ≤ .01

^†^trend-level significance.

**Table 5:** Group differences in metabolic health using IDF clinical-cut off criteria [43].

Variable	Caregivers	Controls	χ2	p
**Baseline**				
*n*	91	91		
Elevated Waist Circumference (%)	81	73	1.98	.22
Elevated Triglycerides (%)	9	2.3	3.81	.10^†^
Decreased HDL Cholesterol (%)	37	18	8.14	.007**
Elevated Blood Pressure (%)	14	13	0.04	1
Elevated Glucose (%)	4.4	3.4	0.13	1
Significant Insulin Resistance (%)	46.7	26.1	10.33	.006**
Metabolic Syndrome (%)	15.7	6.7	3.61	.10^†^
Obesity Status (%)	19.6	16.5	0.29	.70
**2 years**				
*n*	64	73		
Elevated Waist Circumference (%)	89	74	5.05	.03*
Elevated Triglycerides (%)	13	1.4	6.96	.01**
Decreased HDL Cholesterol (%)	24.6	12.9	3	.11
Elevated Blood Pressure (%)	9.8	15.4	1.31	.27
Elevated Glucose (%)	8.2	8.6	0.006	1
Significant Insulin Resistance (%)	55	42.7	1.95	.38
Metabolic Syndrome (%)	14.5	11.4	0.28	.61
Obesity Status (%)	14.1	8.8	1.28	.35

Note.

*p ≤ .05

**p ≤ .01

^†^trend-level significance.

A similar pattern of findings was observed when examining metabolic health cross-sectionally at the 2 year assessment, whereby caregivers had greater levels of insulin (*p* < .001) and LDL cholesterol (*p* = .009), greater waist circumference (*p* = .002), and lower levels of HDL cholesterol (*p* = .02). We observed a trend-level (not statistically significant) pattern of greater WHR among caregivers vs. controls (p = .08; Table 4, bottom panel).The direction and significance of these findings was identical when adjusting for 2 year fat mass rather than BMI. Further, a greater proportion of caregivers, compared to controls, met clinical cut-off criteria for elevated waist circumference (*p* = .03), and elevated triglycerides (*p* = .01; Table 5, bottom panel). Both groups, however, were indistinguishable with regard to all other continuous and clinical cut-off metabolic variables (all *ps*>.11).

**Fig 2:**
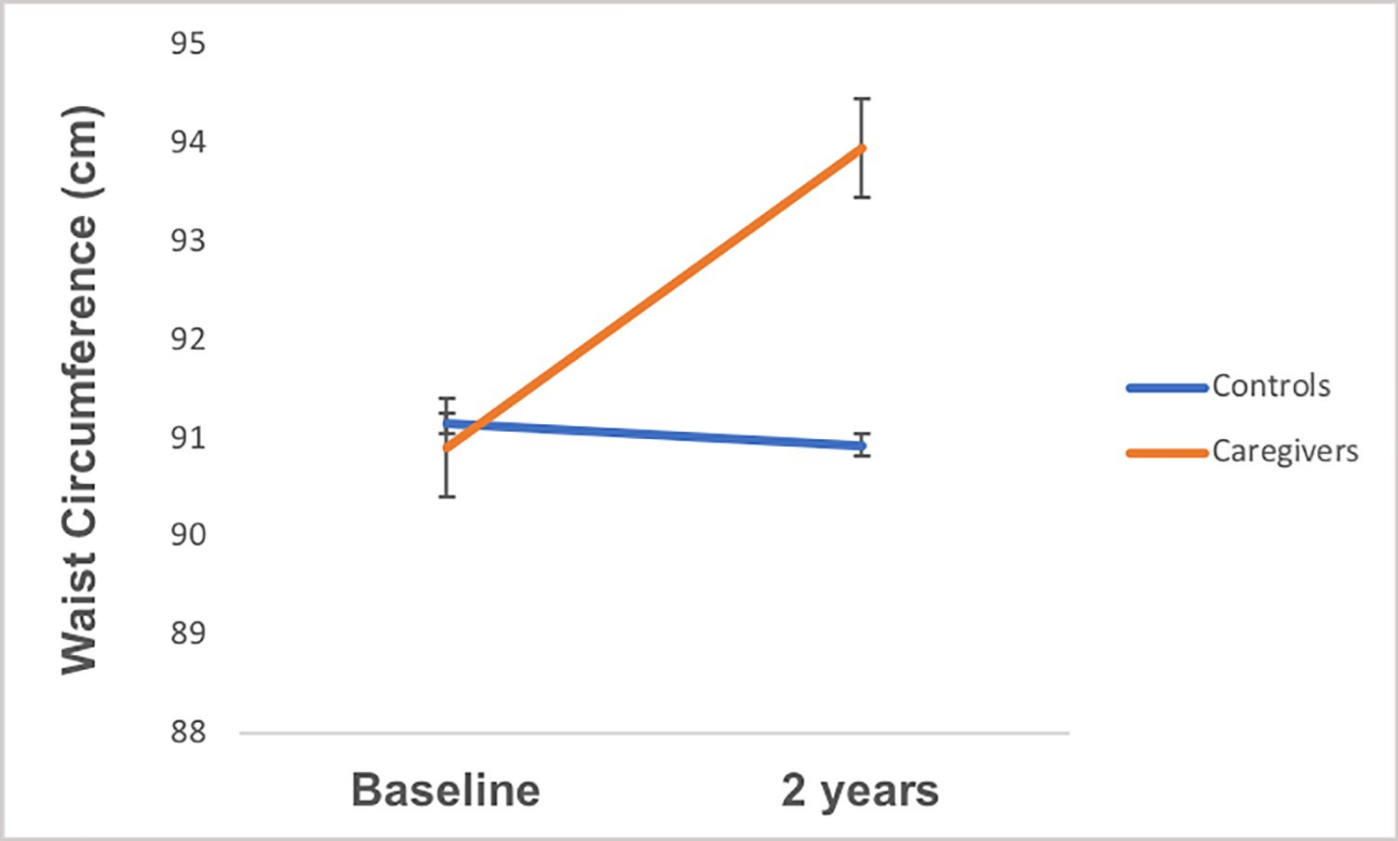
Group differences in waist circumference change from baseline to 2 years, adjusting change in BMI from baseline to 2 years.

**Fig 3:**
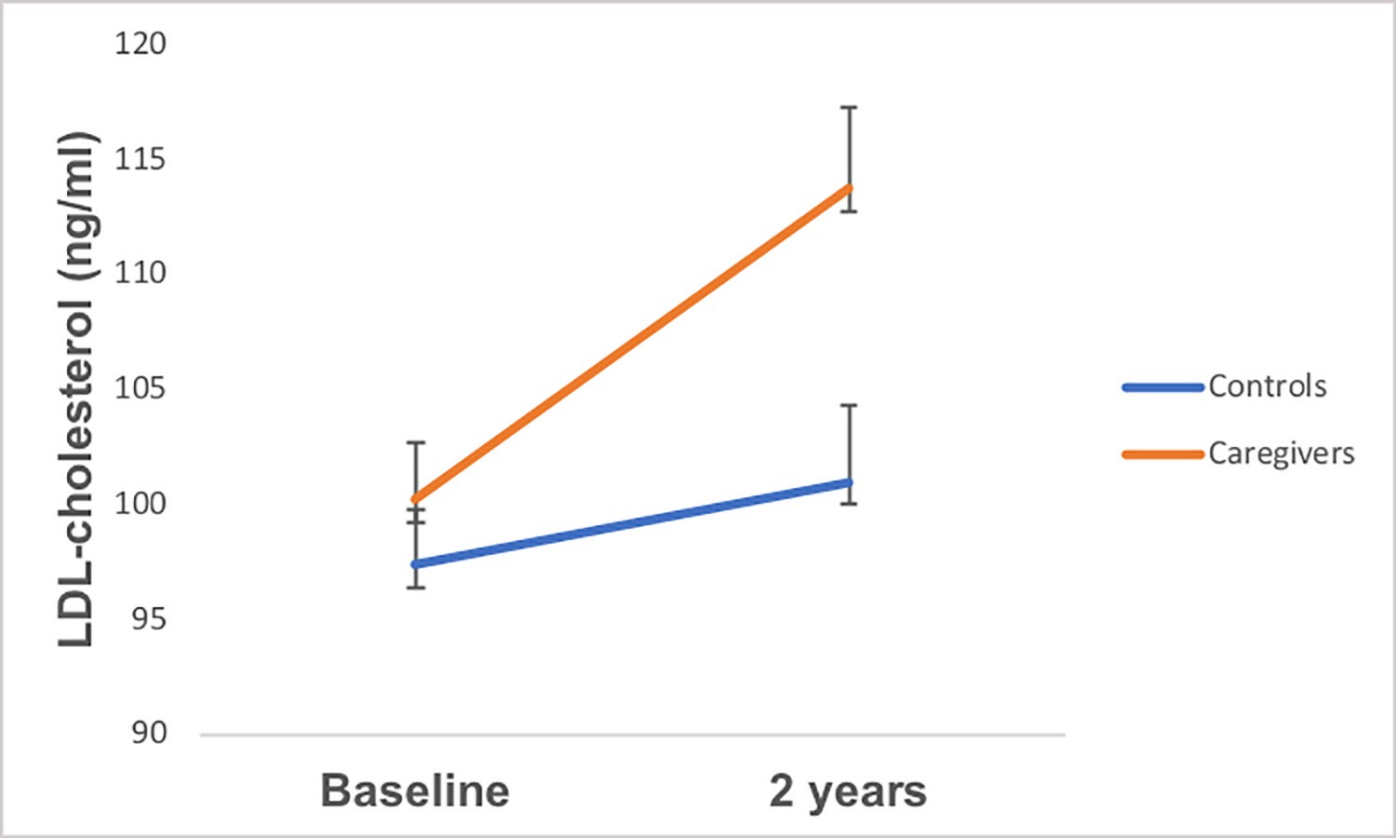
Group differences in LDL-cholesterol change from baseline to 2 years, adjusting change in BMI from baseline to 2 years.

In prospective analyses, caregivers showed significantly greater increases in waist circumference from baseline to 2 years than controls (+3.43 vs. +0.30 cm; *p* < .001; Table 6, bottom panel; Fig 2). The direction and significance of these findings was identical when adjusting for change in fat mass rather than BMI. When adjusting for intervention status, there continued to be a significant main effect of group (*p* < .001), while there were no effects of intervention status, or group X intervention status interactions observed (*ps*>.09). Similarly, the association between group and change in LDL cholesterol approached significance after accounting for the influence of BMI change (*p* = .06; Table 6, top panel; Fig 3). Although not statistically significant, we observed a trend-level pattern of greater increases in LDL cholesterol among caregivers compared to controls from baseline to 2 years (+11.26 vs. +4.94 ng/ml). The direction and significance of these findings was identical when adjusting for change in fat mass rather than BMI. After adjusting for intervention status there was no longer an effect of group (*p* = .10), of intervention status, or group X intervention status interaction (*ps*>.80). Caregivers and controls were indistinguishable with regard to all other continuous metabolic variables in prospective analyses.

**Table 6:** One-way ANCOVA for group differences in metabolic health change from baseline to 2 years, adjusting change in BMI from baseline to 2 years.

	***F***	***p***	***M±SE***_***difference***_	***M±SE***_***difference***_
			Caregivers (n = 59)	Controls (n = 67)
Main effects:				
Δ LDL cholesterol, (ng/ml)	3.62	.06^†^	+11.26 ± 2.42	+4.94 ± 2.27
Covariates:				
Δ BMI	7.6	.007**		
	***F***	***p***	***M±SE***_***difference***_	***M±SE***_***difference***_
			Caregivers (n = 63)	Controls (n = 72)
Main effects:				
Δ Waist circumference, (cm)	17.63	< .001**	+3.43 ± 0.54	+0.30 ± 0.51
Covariates:				
Δ BMI	93.2	< .001**		

*Note*. Unadjusted analyses not presented, as direction and level of significance remained the same in analyses unadjusted for BMI.

*p ≤ .05

**p ≤ .01

^†^trend-level significance.

#### Exploratory analyses

In an effort to understand the observed cross-sectional group differences in metabolic outcomes at each time point, we ran a series of exploratory analyses, first examining correlations between hair cortisol and all metabolic variables of interest at baseline and 2 years, and second, examining moderating role of hair cortisol in the association between group and metabolic outcomes.

Hair cortisol at baseline was unrelated to any metabolic measure at baseline: insulin, glucose, cholesterol (total, HDL, LDL), trigylcerides, SBP/DBP, waist circumference, BMI (all *ps*>.10; Table 3). Identical findings were observed when examining the association between 2 year measures of hair cortisol and metabolic measures.

In a series of moderation analyses at baseline (hair cortisol was interacted with group on the association with baseline metabolic health), we found no main or interaction effects (group X baseline hair cortisol) on fasting insulin (*ps>*.21), leptin (*ps>*.45), HDL cholesterol (*ps>*.25), ghrelin (*ps>*.40), or triglycerides (*ps>*.08). When conducting these same analyses at the 2 year assessment, identical, non-significant findings were observed with regard to the main or interactive effect of 2 year hair cortisol on any metabolic measure.

### Caregiver vs. control differences in eating behaviors

Caregivers, compared to controls, were also distinguished by greater levels of baseline stress-related eating, as measured via the RED-9 (*p* = .05; Table 7, top panel). Similarly, at the 2 year assessment, caregivers had greater eating dysfunction on the RED-9 (*p* = .006; Table 7, bottom panel). In prospective analyses, we found no association between group and change in the RED-9 (*p* = .49). While there was no main effect of group, on average, caregivers increased in RED-9 score from baseline to 2 years by 0.14 points whereas controls increased in RED-9 score by 0.05 points. When including intervention status in the model, we found no main effect of group, intervention status, or group X intervention status interaction (*ps*>.48). When adjusting for intervention status, there continued to be a no effect of group, intervention status, or group X intervention status interaction (*ps*>.40).

**Table 7:** Group differences in reward-driven eating behavior at baseline, adjusting for baseline BMI (top panel); and 2 years, adjusting for 2 year BMI (bottom panel).

Variable	Caregivers	95% CI	Controls	95% CI	df, error	*F*	*p*
	(Adjusted M±SE)		(Adjusted M±SE)				
Baseline							
RED-9^a^	1.12 ± 0.09	(.94, 1.31)	0.87 ± 0.08	(0.69, 1.05)	1, 173	3.65	.05*
2 years							
RED-9	1.28 ± 0.11	(1.05, 1.51)	0.85 ± 0.10	(.65, 1.05)	1, 117	7.83	.006**

*Note*. Unadjusted analyses not presented, as direction and level of significance remained the same in analyses unadjusted for BMI.

*p ≤ .05

**p ≤ .01

^a^RED-9 = Reward-based eating drive scale (Epel et al., 2014)

## Discussion

We found that caregivers of a child with ASD, compared to controls (mothers of a neurotypical child), were distinguished by consistently lower hair cortisol cross-sectionally at baseline and 2 years later. However, group did not differentially predict changes in hair cortisol at 2 years. Indeed, we did not observe significant changes in hair cortisol across our entire sample at 2 years, as well as within each group. Further, caregivers were distinguished, cross-sectionally, by greater reward-related eating at baseline and 2 years, and worse metabolic function at baseline and 2 years (including lower HDL-c and clinically elevated insulin resistance, waist circumference, and triglycerides). In prospective models, caregivers also showed greater increases in waist circumferences at 2 years compared to controls, despite being similar at baseline in BMI and waist circumference. It is particularly noteworthy that this finding was independent of changes in BMI or overall fat mass, suggesting that specific stress-pathways, as opposed to purely obesity-mediated pathways (e.g., due to total calories consumed), may be contributing to worsened metabolic health, specifically greater increases in waist circumference.

Caregiving for a child with a neurodevelopmental disability may represent one of the models of chronic stress that promotes hypocortisolism over time. Our finding of consistently lower hair cortisol levels among high stress caregivers is in accord with a naturalistic study of adults with generalized anxiety disorder [36] and a prospective pilot study of mothers with general caregiving-related stress (compared to mothers reporting low stress; [39]. Furthermore, there is substantial support in the literature for lower (as opposed to higher) cortisol levels among populations experiencing chronic stress and trauma [49–52] (e.g., Heim et al., 2000; Steudte et al., 2013). However, many of these studies used salivary cortisol, as opposed to hair cortisol in their analyses. Further, our results contrast with hypercortisolemia findings (specifically those using hair cortisol) among individuals with a variety of stress-related conditions [26–29, 31], including caregivers of adults with dementia [53].

One potential explanation for this finding of lower levels of cumulative hair cortisol is that increased levels of perceived stress might promote greater initial HPA-axis activity and hypercortisolemia. However, when the presence of a stressor is chronic in nature, compensatory mechanisms might ultimately promote the attenuation of cortisol secretion and subsequent hypocortisolism [49, 54]. Another possibility is that hypocortisolism may in fact promote psychiatric conditions such as heightened anxiety [54], although this is not something that we were able to directly test. While no existing studies have examined hair cortisol among caregivers of youth with ASD specifically, recent studies have found that mothers of an individual with ASD who also reported a greater number of stressful daily life events showed a corresponding flatter diurnal cortisol curve compared to mothers who did not report as many daily stressors [55] and may also demonstrate hyporesponsivity (lower salivary cortisol and immunoglobulin A) to acute stressors [56]. Further, caregivers of children with a serious mental illness have lower daily morning cortisol after stressful days [38]. It is thus possible that a greater accumulation of chronic and daily stressors among caregivers can promote greater allostatic load, as reflected through not only under-responsiveness to acute stressors but also hair cortisol.

Interestingly, hair cortisol at both time points was not directly related to any psychological (e.g., depression, self-reported stress, parental stress) or metabolic factor and most of these variables did not account for why caregivers had lower hair cortisol overall. However, we found a tentative interaction between caregiver group and SES such that among controls, higher levels of SES were associated with higher proximal hair cortisol values, however among caregivers, there was no association between SES and hair cortisol. This may be because there was a restricted range of lower cortisol levels in caregivers. It should be noted, however, that our study was not powered to detect significant interaction/moderation effects. Future research should more carefully examine potential psychological (e.g., anxiety) and metabolic factors that may account for the finding that caregivers have lower hair cortisol.

Our finding that high stress caregivers showed worse metabolic function cross-sectionally and increases in waist circumference prospectively is consistent with prior literature linking chronic stress and metabolic dysfunction in humans [1, 2] and animal models [12]. Similarly, we replicated prior findings of cross-sectional links between chronic stress and greater reward sensitivity and stress-related eating [2–4, 9] among our sample of high stress caregivers. Such findings, in combination with our hair cortisol findings, appear to provide preliminary support for a chronic stress-response network [2, 9] in humans, whereby chronic stress related to caregiving for a child with ASD contributes, in the short-term, to increased acute stress reactivity (as reflected in salivary cortisol), which then promotes greater consumption of highly palatable foods, in order to temporarily decrease negative affect. Over time, a pattern of reward-based eating in response to stress may serve to dampen the HPA-axis reactivity to acute stressors, as seen in animal models [9, 12] and to promote greater waist circumference (due to greater circulating glucocorticoids) and subsequent worsening of metabolic components, such as LDL cholesterol. Future studies should continue to prospectively examine these links and potential mediating or moderating factors.

Given the behavioral and metabolic differences observed between our two groups cross-sectionally, chronically-stressed caregivers of children with ASD may be particularly vulnerable towards worsened health over time. As such, this group may be particularly in need of clinical attention, and may require tailored interventions focusing on malleable factors, such as stress reactivity and appraisal, and reward-based eating, in order to prevent early onset of metabolic disease, including type-2 diabetes and heart disease. For instance, mindfulness-based interventions which focus on reducing stress appraisals and improving eating behavior might promote improvements in mindful eating, decreases in reward-based eating, as well as maintenance in fasting glucose over time [57, 58].

This study had several strengths. To our knowledge, this is the first study to concurrently examine hair cortisol, eating behavior, and metabolic health cross-sectionally and prospectively among high-stress caregivers of children with ASD compared to low-stress caregivers of neurotypical children. Such a longitudinal analysis allows us to make causal inferences about the influence of chronic stress on eating behavior, hair cortisol, and metabolic health. However, our study was not without limitations. Notably, as research utilizing hair cortisol is still in its infancy, the methodology for analyzing and interpreting data is constantly evolving. Researchers have not yet reached a consensus regarding group norms, or potential confounders of hair cortisol, and there may be unknown factors that account for the rate of incorporation of cortisol into the hair such as hair growth rate. For example, we included both a proximal and distal hair segment in our analyses, however the values in the distal hair cortisol samples were consistently lower than those in the proximal hair cortisol samples at each time point. These discrepancies may be related to hair processing (e.g., hair washes) that leech out cortisol. We did not account for this possibility. Additionally, we were unable to assess whether genetic differences potentially accounted for the association between being a caregiver of a child with ASD and cortisol/metabolic function. This is a notable limitation, given that autism is one of the most heritable illnesses, and therefore genetics may play a role in the above associations [59]. Additionally, the added component of an optional 12-week stress-reduction mindfulness-based intervention may have introduced bias in group outcomes. Furthermore, our study suffered from a fair level of attrition over the course of two years, which may have limited our ability to draw stronger conclusions.

## Conclusions

Young, healthy (disease free) parental caregivers are at high risk of early metabolic disease. Although the caregivers and controls were recruited to be similar on BMI, caregivers showed significantly lower hair cortisol levels, and greater reward-based eating, as well as generally worse metabolic functioning as well as greater increases in waist circumference over the course of two years, relative to low-stress caregivers of a neurotypical child. These findings extend the growing literature linking chronic stress to eating behaviors and metabolic health and provide initial support for a chronic stress response network among humans, thus extending the work of Tomiyama and colleagues (2011). We also demonstrated the use of hair cortisol for detecting one phenotype of allostatic load among high-stress caregivers—chronically dampened cortisol output. Caregivers of children with ASD appear to represent a particularly high-risk group for stress-related metabolic disease, and this finding will likely have important clinical implications.

## Supporting information

S1 Table Metabolic syndrome criteria: International Diabetes Federation (IDF). (DOCX)

## Abbreviations

HDL: High-density lipoprotein
LDL: Low-density lipoprotein
TGL: Triglycerides
HOMA-IR: Homeostatic model of insulin resistance
BMI: Body mass index
ASD: Autism spectrum disorder
PSS: Perceived stress scale
