# Correction: Maternal caregivers have confluence of altered cortisol, high reward-driven eating, and worse metabolic health

**DOI:** 10.1371/journal.pone.0221354

**Published:** 2019-08-14

**Authors:** Rachel M. Radin, Ashley E. Mason, Mark L. Laudenslager, Elissa S. Epel

In the Author Contributions, Rachel M. Radin (RMR) should be listed as one of the persons who conceptualized the study.
