# Supplementary material for: Maternal caregivers have confluence of altered cortisol, high reward-driven eating, and worse metabolic health
Source: PLoS One. 2019 May 10;14(5):e0216541. doi: 10.1371/journal.pone.0216541 (PMC6510426; doi:10.1371/journal.pone.0216541)
Supplement: S1 Table — (DOCX) [file pone.0216541.s001.docx]

|  | **Clinical cut-off criteria** |  |
| --- | --- | --- |
| *Metabolic Syndrome presence defined when a participant meets cut-off criteria for at least 3 of the following factors, one of which must include elevated waist circumference* | | |
| *Waist Circumference | >80 cm |  |
| Raised Triglycerides | ≥ 150 mg/dL | Or specific treatment for this condition |
| Reduced HDL Cholesterol | <50 mg/dL | Or specific treatment for this condition |
| Raised Blood Pressure | ≥ 130 mm Hg for SBP,  or ≥ 85 mm Hg for DBP | Or specific hypertension treatment |
| Raised Fasting Plasma Glucose | ≥ 100 mg/dL | Or previously diagnosed type 2 diabetes |
| Insulin Resistance  (HOMA-IR) | >1.9 = early insulin resistance  >2.9= significant insulin resistance |  |
